# Stress and Eating Behavior: A Daily Diary Study in Youngsters

**DOI:** 10.3389/fpsyg.2018.02657

**Published:** 2018-12-21

**Authors:** Taaike Debeuf, Sandra Verbeken, Marie-Lotte Van Beveren, Nathalie Michels, Caroline Braet

**Affiliations:** ^1^Department of Developmental, Personality and Social Psychology, Ghent University, Ghent, Belgium; ^2^Department of Public Health, Ghent University, Ghent, Belgium

**Keywords:** daily diary, stress, eating behavior, emotion regulation, emotional eating

## Abstract

**Background:** Overweight and obesity are growing problems, with more attention recently, to the role of stress in the starting and maintaining process of these clinical problems. However, the mechanisms are not yet known and well-understood; and ecological momentary analyses like the daily variations between stress and eating are far less studied. Emotional eating is highly prevalent and is assumed to be an important mechanism, as a maladaptive emotion regulation (ER) strategy, in starting and maintaining the vicious cycle of (pediatric) obesity.

**Objectives:** The present study aims to investigate in youngsters (10 – 17 years) the daily relationship between stress and the trajectories of self-reported eating behavior (desire to eat motives; hunger eating motives and snacking) throughout 1 week; as well as the moderating role of emotion regulation and emotional eating in an average weight population.

**Methods:** Participants were 109 average weighted youngsters between the age of 10 and 17 years (*M*_age_ = 13.49; *SD* = 1.64). The youngsters filled in a trait-questionnaire on emotion regulation and emotional eating at home before starting the study, and answered an online diary after school time, during seven consecutive days. Desire to eat motives, hunger eating motives and snacking were assessed daily for seven consecutive days.

**Results:** Using multilevel analyses results revealed that daily stress is significantly associated with trajectories of desire to eat motives and hunger eating motives. No evidence was found for the moderating role of maladaptive ER in these relationships; marginally significant evidence was found for the moderating role of emotional eating in the trajectories of desire to eat and snacking.

**Discussion:** These results stress the importance of looking into the daily relationship between stress and eating behavior parameters, as both are related with change over and within days. More research is needed to draw firm conclusion on the moderating role of ER strategies and emotional eating.

## Introduction

Pediatric overweight and obesity are growing problems, as the prevalence of obesity is tripled since 1975. Worldwide, 18% of the children and adolescents (5–19 years) are diagnosed with overweight and 8% with obesity (World Health Organization [WHO], 2018). Parallel to the global rise in pediatric obesity, metabolic, psychological and social health problems are beginning to appear in childhood; and positive associations between these phenomena are found (Braet et al., 1997; Zipper et al., 2001; Reilly et al., 2003; Erermis et al., 2004; Lobstein et al., 2004; Reilly and Kelly, 2011). Moreover, these diseases are known to track into adulthood (Ferraro and Kelley-Moore, 2003; Reilly et al., 2003; Lifshitz, 2008; Reilly and Kelly, 2011). This progress and the assumed comorbidities emphasize the importance of studying the underlying mechanisms leading to childhood obesity. Psychological and psychophysiological research in this field emphasizes the influence of stress on eating and weight regulation.

### Stress Conceptualization

Psychological stress occurs when a person appraises a situation as significant for his or her welfare and when the situation exceeds his or her available coping resources (Lazarus and Folkman, 1986). This definition implicates a subjective component that can be captured only through self-report (Krohne, 2002). Besides, the emotional value of the stressor needs to be taken into account. Lazarus (1993) states that stress is closely related to the conceptualization of emotions because higher levels of stress are consistently associated with higher negative affect but so far, specifically in childhood, this link is less studied in the context of emotional eating (Cohen et al., 1995; Watson, 1998; Lemyre and Tessier, 2003; Mroczek and Almeida, 2004; Minkel et al., 2012; Gustafsson et al., 2013; Bey et al., 2018).

As our stress levels know day-level fluctuations within persons (Beattie and Griffin, 2014), these should be studied as ‘daily hassles,’ which are situations, thoughts or events leading to negative feelings (e.g., annoyance, irritation, worry or frustration) when they occur and inform you on the difficulty or impossibility to achieve your goals and plans (O’Connor et al., 2008).

Stress is associated with the development of different medical and psychological disorders (Salim, 2014). Pertinent to the current study, research in children and adolescents proved that the experience of negative affect and stress is associated with weight gain, a higher waist circumference, a higher BMI and in the long term obesity (Goodman and Whitaker, 2002; Koch et al., 2008; De Vriendt et al., 2009; Midei and Matthews, 2009; Rofey et al., 2009; Van Jaarsveld et al., 2009; Aparicio et al., 2016).

### Stress and Eating Behavior

Psychological research shows that stress is associated with overweight and obesity through changes in weight-related health behavior, as stress activates emotional brain networks and elevates the secretion of glucocorticoids and insulin. Both the emotional brain networks and the hormones influence different aspects of our eating behavior such as our food intake, food choice and eating motives (hunger and desire eating) (O’Connor et al., 2008; Dallman, 2010; Jastreboff et al., 2013).

First, regarding food intake, stress may result in under- or overeating depending on the stress source and stress intensity (Willenbring et al., 1986; Steere and Cooper, 1993; O’Connor et al., 2008; Ansari and Berg-Beckhoff, 2015). Concerning undereating, adults and children are often found to eat less after heavy stressful events and, specifically, family stress is associated with underweight (BMI) (Popper et al., 1989; Stone and Brownell, 1994; Oliver and Wardle, 1999; Stenhammar et al., 2010). However, evidence for overeating after experiencing stress is also found. Next to the detected underweight after family stress, Stenhammar et al. (2010) also report possible overweight after family stress, suggesting a link with respectively under- and overeating. Besides, Evers et al. (2010) and Vandewalle et al. (2017b) show in youngsters how induced negative affect leads to an increased food intake, specifically comfort foods.

Second, concerning food choice, stressor intensity is an important factor. In adults, chronic life stress is associated with the intake of more energy-dense food (Steptoe et al., 1998; Torres and Nowson, 2007; O’Connor et al., 2008) a lower consumption of main meals and vegetables (O’Connor et al., 2008). In a systematic review and meta-analysis on children, Hill et al. (2018) report that stress, measured via self-reports and via cortisol measures, is associated with the intake of more unhealthy and less healthy food items (8–18 years).

Third, concerning eating motives, De Vriendt et al. (2009) conclude in their review that in adolescents, stress is specifically associated with increased appetite, which can be seen as a desire for food. Therefore, a distinction should be made between ‘hunger eating motives’ (=eating out of hunger) and ‘desire to eat motives’ (=eating out of a desire to eat; eating out of a craving for food), with the latter defined as rather unhealthy eating (Reichenberger et al., 2016, 2018). Here, a recent EMA study in adults finds that time pressure is associated with more hunger eating (Reichenberger et al., 2016). Goldschmidt et al. (2018) shows in the only available ecologically momentary assessment study (EMA = looking into the current real time behaviors) in obese children in their natural environment that specifically ‘desire to eat motives’ were of importance regarding overeating.

These ecological momentary assessment results (O’Connor et al., 2008; Reichenberger et al., 2016) stress the importance of looking into the daily fluctuations in within-person stress levels to better understand the precise role of different daily stressors in eating behavior, on top of the existing measurements of stress. Besides, as eating behaviors are a daily occupation, and appear in different contexts, a diary study can further help to capture the relationship between daily hassles and different indicators of eating behavior.

However, this research in children and adolescents is lacking, but highly needed and relevant as (1) adolescence is an important developmental stage (Giedd et al., 2009); (2) Goldschmidt et al. (2018) showed the importance of daily desire to eat motives in obese youngsters and (3) daily stress is positively associated with daily fluctuations in scores on emotional eating (Vandewalle et al., 2017a). It is hypothesized that stress- induced eating or emotional eating, defined as “eating your negative emotions away,” can be seen as a maladaptive emotion regulation (ER) strategy in children also, but this remains to be further explored (Braet and Van Strien, 1997; Thayer, 2001; Evers et al., 2010).

### Role of Emotion Regulation Processes

#### Emotion Regulation Strategies

Emotion regulation refers to the actions by which persons try to influence their emotions, more specifically which emotions they experience, when and how they experience the emotions and how they show their emotions to others (Gross, 1998). Emotion regulation strategies are studied most often in contexts in which persons upregulate their (stress related) negative emotions. Emotion regulation strategies influence eating behavior and health behavior (Whiteside et al., 2007; Evers et al., 2010; Koenders and Van Strien, 2011), and weight gain and obesity (De Vriendt et al., 2009). This association is stronger when maladaptive emotion regulation strategies are used to deal with the stressor in comparison with the use of adaptive emotion regulation strategies (Aparicio et al., 2016). In a longitudinal perspective, toddlers with lower levels of emotion regulation skills (i.e., more negative emotion reactions and less adequate emotion regulation) have more overweight at the age of 10 (Graziano et al., 2014), suggesting that difficulties in emotion regulation skills in 2 until 5 years old is related to the development of pediatric obesity later in life (Graziano et al., 2010). Using maladaptive emotion regulation strategies is found to mediate the association between the stressor of maternal rejection and emotional eating (measured as a trait, with the DEBQ-questionnaire) in obese youngsters (Vandewalle et al., 2014). On the other hand, adaptive emotion regulation strategies are related with positive health behaviors such as a higher intake of fruits and vegetables and more physical activity (Isasi et al., 2013). Remarkably, whether people with high/low maladaptive emotion regulation strategies cope differently with stress is less studied.

#### Emotional Eating as Emotion Regulation Strategy

Emotional eating is highly prevalent in community-wide youth samples (Michels et al., 2012) and in youth with obesity (Braet and Beyers, 2009). Besides, emotional eating is treatment resistant, as it often persists after obesity treatment (Vandewalle et al., 2018). Emotional eating can occur in the presence of a lot of different emotions (Faith et al., 1997) and again, is most studied regarding stress-related emotions. In children, high scores on emotional eating have been associated with both negative feelings on physical competencies (Braet and Van Strien, 1997) and experienced stress (Nguyen-Rodriguez et al., 2009; Michels et al., 2012). Emotional eating has a short term reinforcing effect, by reducing stress-related arousal and negative affect (Popless-Vawter et al., 1998; Macht, 2008). However, in the long-term using the strategy of ‘emotional eating’ in particular in obese persons, often instills feelings of guilt and self-anger (Popless-Vawter et al., 1998), thereby intensifying the existing negative emotions (Popless-Vawter et al., 1998; Gibson, 2006). Even more important, emotional eating will not solve the stress origins: as long as people only cope with their stress by means of emotional eating, the stressor will remain and cause psychological discomfort (Stice et al., 2002; Gibson, 2006). This way, emotional eating can be seen as a proxy of maladaptive emotion regulation (Michopoulos et al., 2015).

Emotional eating is associated with more calorie-intake (Braet and Van Strien, 1997). This is of concern as emotional eating seems to evolve toward a stable trait component later in life and is of greater importance compared with other life style behaviors for explaining longitudinal weight gain (Koenders and Van Strien, 2011). Persons with a high emotional eating style who are confronted with a stressor eat more high-fat food and more energy-dense meals in comparison with persons who have a low emotional eating style (Oliver et al., 2000). O’Connor et al. (2008) concludes in an EMA-study that the confrontation with daily hassles is associated with more snacking, and this is stronger for individuals who have a high emotional eating style. Interesting, the presence of negative emotions decreases the desire eating in individuals with a low emotional eating style; while the negative emotions do not affect the desire eating in individuals with a high emotional eating style (Reichenberger et al., 2016). This suggests that emotional eating evolves toward a stable trait that might be a moderator in the stress-eating relation.

### Research Questions and Daily Diary-Design

**FIGURE 1:**
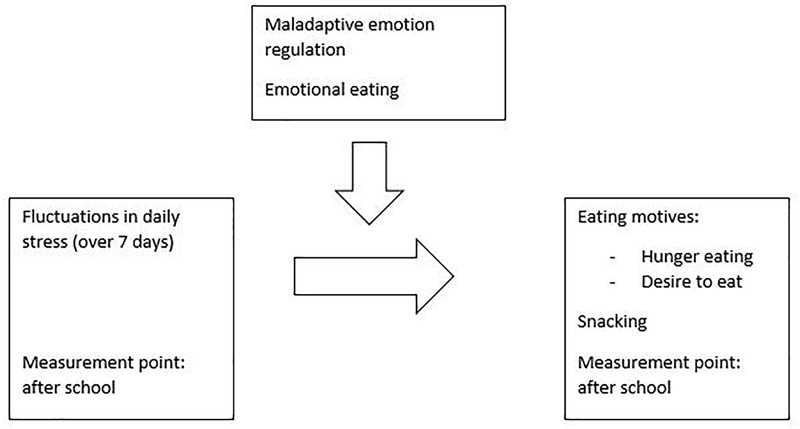
Research question.

The current study is innovative in different ways: (1) by including children and adolescents, (2) by using a daily diary design during seven consecutive days and (3) by including three indicators of eating behavior; daily hunger eating motives, desire to eat motives and snacking; and their moderating factors, emotion regulation and emotional eating. However, as using a cellphone during school time is often forbidden, the eating motives and snacking during the day will be reported after school time, which is a well-known critical phase with both school related and peer related stress (Sotardi, 2017). The research question is shown in Figure 1.

First, we hypothesize that emotional eating will be a proxy of maladaptive emotion regulation (based on Michopoulos et al., 2015). Second, we state that daily stress will be significantly associated with (1) the trajectories in desire to eat (2) the trajectories of hunger-eating and (3) the trajectories of snacking (Based on De Vriendt et al., 2009; Reichenberger et al., 2016; Vandewalle et al., 2017a). We also hypothesize that these relations will be stronger for youth high in trait maladaptive emotion regulation and/or reporting a more pronounced emotional eating style (O’Connor et al., 2008; Reichenberger et al., 2016).

## Methodology

### Participants

Dutch- speaking children and adolescents were recruited in Belgium, Aalter and Deinze. These youngsters already participated in two previous studies: Generation 2020 study (Van Beveren et al., 2016, 2017) and the Reward- study (De Decker et al., 2017a,b). These are both longitudinal studies with different data collection waves over time on the emotional wellbeing of children and adolescents. During a follow-up data collection in both cohorts, participants were recruited for the current study. A total of 109 participants aged 10–17 years were recruited [*M*_age_ = 13.49; *SD* = 1.64; 51 boys (46.8%) and 57 girls (52.3%)]; one participant did not report their age and gender.

### Study Design

**FIGURE 2:**
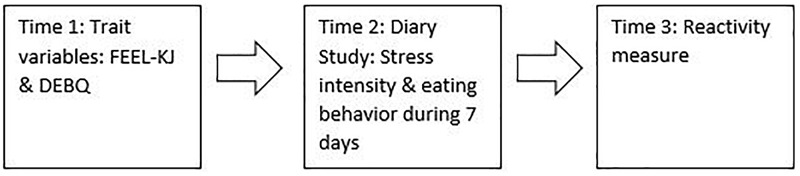
Measurements.

Before the diary study, the participants were asked to fill out questionnaires on eating behavior and emotion regulation. Following this, the participants were required to complete the stress/eating behavior diary over seven consecutive days. EMA measures were similar to the study of Reichenberger et al. (2016), but extended by adding the variable of snacking and the role of emotion regulation (Figure 2).

The diary was filled out before breakfast, after school time, when the participants came home from school and before bedtime. In the current study, only the data time point after school was used, which was in most cases filled in between 5 PM and 6 PM. At 5 PM the participants received a reminder message to fill out the diary. Participants were asked to report on their snacking behavior since breakfast.

#### Trait Variables by Questionnaires

##### FEEL-KJ

The Fragebogen Zur Erhebung der Emotionsregulation Bei Kindern und Jugendlichen (FEEL-KJ, Grob and Smolenski, 2005; A Questionnaire on Emotion Regulation Strategies in Children and Adolescents) is translated from German to Dutch and can be used in children and adolescents between 8 and 18 years old (Braet et al., 2013). The questionnaire measures ER strategies for three emotions: anger, anxiety and sadness. The emotion regulation strategies for every emotion are captured by 30 items, thus the total amount of items is 90. The 15 emotion regulation strategies are divided in three categories of strategies: adaptive, maladaptive and external regulation. The adaptive emotion regulation strategies are distraction, recall a positive emotion, forget, acceptance, problem solving, re-evaluation of the situation, and cognitive reappraisal. The maladaptive strategies are giving up, withdrawing, self- devaluation, rumination and aggression. Looking for social support, controlling your emotions and expressing your emotions are the external strategies. For the Dutch and Flemish population, representative norms are available (Braet et al., 2013). The psychometric qualities of the FEEL-KJ are drawn from a large sample of Dutch speaking Belgian children and adolescents between 8 and 18 years old. A good reliability and validity was found by Cracco et al. (2015). In the current study, a good reliability was also found for both subscales adaptive and maladaptive emotion regulation, respectively α = 0.957 and α = 0.831.

##### Dutch Eating Behavior Questionnaire

The Dutch Eating Behavior Questionnaire (DEBQ, van Strien et al., 1986; Braet et al., 2008) assesses three eating styles: restrained eating, external eating and emotional eating. In this study only the subscale ‘emotional eating’ will be taken into account. The DEBQ contains 33 items, and is a self-report questionnaire. The items question specific eating behaviors, and should be rated on a five-point- Likert scale from 1 = never to 5 = very often. The DEBQ has been shown useful in research with children and adolescents between 7 and 17 years (Braet et al., 2003). Also, good psychometric qualities are reported, such as a good reliability and external validity (Ricciardelli and McCabe, 2001; Braet et al., 2008). The emotional eating subscale contains 13 items and has a good internal validity in overweight and normal-weight children (respectively α = 0.91 and α = 0.93) (Braet et al., 2008). In the current study, a good reliability for emotional eating was found (α = 0.946).

#### Diary-Measures

##### Stress intensity

To measure the amount of stress during a particular school day, the participants were asked to rate ‘how stressed did you feel since your breakfast?’ on a visual analog scale from 0 (not at all stressed) to 100 (extremely stressed). By asking about the period since breakfast until after school, we wanted to capture stress related to school performances and peer-related stress (social stress).

##### Snacking and motives of eating behavior

The after school diary questions always start with the question ‘How many snacks did you eat since breakfast?’. A snack was defined as ‘healthy and unhealthy food and/or drinks, which were not part of your main meals, e.g., lunch’. Participants had the option of choosing between ‘0 or 1,’ ‘2,’ ‘3,’ and ‘4.’ For every snack, they needed to fill out what they ate as a snack and how many, e.g., 1 apple or 1 large snicker. Using the Nobelguide, two researchers scored and discussed until agreement on the amount of Kcal, and this was double checked by a dietitian, in the case of non-accordance. For every reported snacks, the hunger eating motive and the desire to eat motive was measured using the following questions: ‘rate the extent to which you agree with the following statement of “I am hungry”’ and ‘rate the extent to which you agree with the following statement of “I desire to eat”.’ Both statements were rated on a 5-point Likert scale with 1 = strongly disagree and 5 = strongly agree. In the analysis, a calculated mean score on the amount of snacks (kcal) and a calculated mean score on hunger and desire eating motives was used.

##### Control variables

As control variables, age and gender were taken into account.

#### Reactivity Measures

It is important to know if the act of completing the daily diary impacted the behavior of the participant (reactivity, e.g., if the participants knew they had to fill out all the questions related to every snack, they might have eaten fewer snacks than normal). Therefore, at the end of the current study, reactivity was measured with a single question regarding the influence the diary had on their eating behavior. The responses were rated on a 7-point Likert scale with 1 = I ate much less, 2 = Sometimes I ate less, 3 = I ate a little bit less, 4 = filling in the diary had no effect on my eating behavior, 5 = I ate a bit more, 6 = sometimes I ate more and 7 = I ate much more.

### Procedure

Children and adolescents between 10 and 17 years old were recruited as part of a larger research project (*Emotion Regulation in the prevention and treatment of obesity in young adolescents).*

When participants and their parents agreed on participating in the diary-study, a home visit was planned. During the first home visit the informed consents were signed by the youngsters and their parents, the trait questionnaires were explained and a smartphone was given to the participants to fill out the diary. Together with them, an example of 1 diary assessment point was filled out. Participants had to fill out the diary for 7 days, with the assessment points after breakfast, after school and before bedtime. In the current study, only the measurement point after school time is used to answer the research questions. Collecting data during this limited window of time, seven consecutive days, was based on other recent studies in youth (Hruska et al., 2017; Vandewalle et al., 2017a; Mabbe et al., 2018).

During the diary- data collection, the participants received at every assessment point an e-mail with the link to the diary questions as a reminder for filling in the diary. To log in on the website, every participant received a unique code, which guaranteed the anonymity of the participants. The diary questions were also given in a written format; in case the online version would give problems. When the participant did not fill in the diary three consecutive times, e-mails with the links were not sent any more to this person. At the fourth day of the diary-study, the participants received a phone call to ask how the study went, if there were difficulties and to encourage them to carry on until day 7 including an integrity check whereby the snack registration of that day was asked.

After the study, a second home visit was planned to collect the trait questionnaires and smartphones. During the second home visit, the reactivity measure was asked to the participants; and they received a film ticket as reward.

This study was approved by the Ethical Committee of Ghent University.

### Data-Analysis

Data were analyzed within a multilevel framework using MLwiN (Rasbash et al., 2009) because the seven consecutive days (Level 1) were nested within the individuals (Level 2). In following Nezlek (2011) recommendations, variables at Level 1 reflecting within-youth predictors were centered around the individual’s mean (group-mean centered; Enders and Tofighi, 2007; Nezlek, 2011) in order to eliminate the influence of Level 2 differences in the predictors for the analyses. Predictors at Level 2 reflecting between-youth predictors were centered around the grand mean (grand-mean centered) to improve the interpretation of the intercept values (Nezlek, 2011).

As proposed by Singer and Willet (2003), we fitted two initial models, namely the random intercept-only model and the unconditional growth model for daily desire to eat, hunger eating, and snacking prior to our main analyses. First, random-intercept-only models (Null Models) were used to estimate the variance partitioning coefficient (VPC), which reflects the proportion of variance in the dependent variable situated between youth (Level 2). In a second step, unconditional growth models (Singer and Willet, 2003) were built stepwise from the Null Models by adding Time as a Level 1 predictor, reflecting whether on average there was change over time. Furthermore, we tested whether the growth rate varied between individuals by modeling the coefficient of Time random at Level 2 (i.e., random slope; unconditional growth models). The likelihood ratio test (LRT) was used to compare the goodness of fit of nested models. To evaluate the significance of the fixed and random effects the Wald test was used. Gender, age, and adjusted BMI were included as covariates of no interest in all undermentioned analyses. To test whether daily stress and trait maladaptive emotion regulation interact in explaining trajectories of desire to eat, hunger eating, and snacking respectively we extended the baseline growth models for these variables by adding daily stress, trait maladaptive emotion regulation, and their interaction to the unconditional growth models.

## Results

Throughout the week, a decreasing compliance for filling out the online diary was observed, with respectively 96, 83, and 77 youngsters filling out the diary at day 1, day 4, and day 7.

Means, standard deviations, and correlations between all variables are shown in Table 1. Mean scores of all variables in the current sample are comparable to findings of previous studies in youth samples (Braet and Beyers, 2009; Braet et al., 2013). When comparing the mean scores for emotional eating and emotion regulation with the gender- and age specific norms of the trait questionnaires (DEBQ and FEEL-KJ), all mean scores are not significantly different from the norm scores; except for the use of maladaptive emotion regulation strategies in boys between 13 and 17 years old [*t*(32) = 3.09; *p* < 0.01] (Table 2; Braet and Beyers, 2009; Braet et al., 2013). Comparing the daily mean score for kcal-intake of snacking with previous research in girls and boys between 9 and 14 years (Field et al., 2004), the mean score was significantly lower from the mean-score in their research.

**Table 1:** Comparison of means with gender- and age specific norm scores.

Group	Variables	*M* (*SD*) in participants of current research	*M* (*SD*) norm scores	*T*-score	Df	*p*-value
(1) Boys 10–12 years	Emotional eating	2.36 (0.93)	2.27 (0.69)	0.403	16	0.693
	Adaptive ER	123.25 (32.70)	133.8 (28.9)	-1.290	15	0.216
	Maladaptive ER	73.31 (9.47)	71.8 (15.6)	0.639	15	0.533
(2) Boys 13–17 years	Emotional eating	2.07 (0.69)	1.98 (0.73)	0.564	33	0.577
	Adaptive ER	133.52 (29.94)	140.4 (26.5)	-1.319	32	0.197
	Maladaptive ER	76.58 (14.26)	68.9 (15.2)	3.094	32	<0.01^∗∗^
(3) Girls 10–12 years	Emotional eating	2.28 (0.69)	2.20 (0.63)	0.483	16	0.636
	Adaptive ER	137.13 (19.80)	138.4 (27.2)	-0.258	15	0.800
	Maladaptive ER	72.81 (11.11)	75.2 (14.6)	-0.859	15	0.404
(4) Girls 13–17 years	Emotional eating	2.35 (0.76)	2.20 (0.78)	1.254	39	0.217
	Adaptive ER	131.68 (24.37)	136.1 (23.9)	-1.117	37	0.271
	Maladaptive ER	82.01 (12.41)	79.0 (17.6)	1.497	37	0.143

*ER, emotion regulation. ^∗∗^*p* < 0.01.*

**Table 2:** Variable correlations and descriptives.

Variables	1	2	3	4	5	M (*SD*)	Min	Max
(1) FEEL-KJ Maladaptive ER						77.49 (12.85)	47.00	119.00
(2) DEBQ emotional eating	0.304^∗∗^					29.27 (10.62)	13.00	65.00
(3) Daily stress	0.162	0.061				13.57 (15.78)	0	84.29
(4) Daily desire eating	0.046	0.100	0.267^∗∗^			2.29 (1.12)	0.14	4.43
(5) Daily hunger eating	-0.042	0.006	0.274^∗∗^	0.889^∗∗^		2.30 (1.10)	0.14	4.71
(6) Daily snacking	-0.033	0.029	0.118	0.664^∗∗^	637^∗∗^	152.83 (95.87)	0	405.97

*ER, emotion regulation; Daily measures = mean of daily measures over the 7 consecutive days ^∗∗^*p* < 0.01.*

Maladaptive emotion regulation was positively correlated with emotional eating, both measured as trait variables (*r* = 0.304, *p* < 0.01) confirming the presumption that emotional eating functions as a proxy of maladaptive emotion regulation. Additionally, all daily measurements were significantly and positively correlated, except for daily stress with daily snacking. Higher levels of daily stress were significantly associated with higher levels of daily desire to eat and hunger eating (*r* = 0.267 and *r* = 0.274, *p* < 0.01, respectively) and all three indicators of eating behavior were significantly correlated (Table 1). Concerning the hunger eating motives and desire to eat motives, the multicollinearity was checked by the variance inflation factor (VIF). With VIF = 1.00, it was concluded that both constructs did not overlap substantially, and thus multicollinearity was not a problem.

### Reactivity Measure

Concerning the reactivity measures, 98 participants filled out this question at the second home visit. Unfortunately, in 11 participants the reactivity measure was missing, due to the fact that the participants were not at home at the time of the second home visit. On the 7-point Likert scale the minimum score indicated by the participants was 2 (“Sometimes I ate less due to filling out the diary”) and the maximum score 5 (“I ate a little bit more due to filling out the diary”), respectively by 4 (3.7%) and 2 (1.8%) participants. The major group of participants stated that “filling out the diary had no influence on their snacking behavior” (Likert-score 4) (*n* = 82; 75.2%), and 9.2% (*n* = 10) stated that “they ate a little bit less due to filling out the diary” (Likert-score 3).

### Between and Within Youth Variance of Stress and Self-Reported Eating Behavior

We first fitted random intercept-only models (i.e., Null Models) for core study variables as a baseline for more complex models. Variance estimates and standard errors (SE) are depicted in Table 3 (desire to eat), Table 4 (hunger eating), and Table 5 (snacking). For all variables, the between-youth and within-youth variance components were significant. The VPC indicated that 85.08% of the variance in daily desire to eat, 84.86% of the variance in daily hunger eating and 80.87% of the variance in daily snacking was situated between youth. Before running the main analyses, we fitted unconditional growth models for core study variables. These models revealed that levels of desire to eat, hunger eating, as well as snacking generally decreased as the week progressed.

**Table 3:** Daily desire to eat as a function of stress, maladaptive emotion regulation, and trait emotional eating.

	Null Model	Model 1	Model 2	Model 3
**Fixed parameters**				
Constant	2.69 (0.09)^∗∗∗^	2.82 (0.10)^∗∗∗^	2.81 (0.10)^∗∗∗^	2.81 (0.10)^∗∗∗^
Time		-0.05 (0.02)^∗^	-0.26 (0.17)	-0.23 (0.17)
**Trajectories**				
Age × time			0.02 (0.01)	0.01 (0.01)
Gender × time			0.01 (0.04)	-0.01 (0.04)
adjBMI × time			-0.01 (0.01)^∗^	-0.01 (0.01)^∗^
Stress × time			0.01 (0.01)^∗∗∗^	0.01 (0.01)^∗∗∗^
ER × time			-0.01 (0.01)	
Stress × ER × time			-0.00 (0.00)	
EE × time				0.01 (0.01)^†^
Stress × EE × time				0.00 (0.00)
**Random Parameters**				
$\sigma_{\mathrm{u0}}^{2}$	0.66 (0.11)^∗∗∗^	0.68 (0.14)^∗∗∗^	0.66 (0.14)^∗∗^	0.67 (0.14)^∗∗∗^
σ_u0u1_		-0.03 (0.02)	-0.04 (0.02)	-0.05 (0.03)^∗^
$\sigma_{\mathrm{u1}}^{2}$		0.02 (0.01)^∗∗^	0.02 (0.01)^∗∗^	0.02 (0.01)^∗∗∗^
$\sigma_{\mathrm{e0}}^{2}$	0.61 (0.04)^∗∗∗^	0.50 (0.04)^∗∗∗^	0.49 (0.04)^∗∗∗^	0.49 (0.04)^∗∗∗^
**Deviance**	1434.28	1403.49	1385.01	1382.96

*The reference category for gender was male. adjBMI, adjusted BMI; ER, maladaptive emotion regulation; $\sigma_{\mathrm{u0}}^{2}$, between-person variance; $\sigma_{\mathrm{u1}}^{2}$, variance in the slope; $\sigma_{\mathrm{e0}}^{2}$, within-person variance; σ_*u0u1*_, covariance between random intercept and random slope; Null Model, random intercept-only model for desire to eat; Model 1, unconditional growth model for desire to eat. ^∗^*p* < 0.05, ^∗∗^*p* < 0.01, ^∗∗∗^*p* < 0.001; ^†^marginally significant: *p* < 0.10.*

**Table 4:** Daily hunger eating as a function of stress, maladaptive emotion regulation, and trait emotional eating.

	Null Model	Model 1	Model 2	Model 3
**Fixed parameters**				
Constant	2.74 (0.09)^∗∗∗^	2.98 (0.10)^∗∗∗^	2.97 (0.10)^∗∗∗^	2.97 (0.10)^∗∗∗^
Time		-0.09 (0.02)^∗∗∗^	-0.09 (0.15)	-0.05 (0.14)
**Trajectories**				
Age × time			-0.00 (0.01)	-0.01 (0.01)
Gender × time			0.01 (0.04)	0.01 (0.03)
adjBMI × time			-0.01 (0.01)	-0.00 (0.01)
Stress × time			0.01 (0.01)^∗∗^	0.01 (0.01)^∗∗^
ER × time			-0.01 (0.01)	
Stress x ER × time			0.00 (0.00)	
EE × time				0.01 (0.01)
Stress × EE × time				-0.00 (0.00)
**Random Parameters**				
$\sigma_{\mathrm{u0}}^{2}$	0.62 (0.11)^∗∗∗^	0.52 (0.12)^∗∗∗^	0.51 (0.12)^∗∗∗^	0.51 (0.12)^∗∗∗^
σ_u0u1_		0.02 (0.02)	0.02 (0.02)	0.02 (0.02)
$\sigma_{\mathrm{u1}}^{2}$		0.01 (0.01)	0.01 (0.01)	0.01 (0.01)
$\sigma_{\mathrm{e0}}^{2}$	0.63 (0.04)^∗∗∗^	0.58 (0.04)^∗∗∗^	0.56 (0.04)^∗∗∗^	0.56 (0.04)^∗∗∗^
**Deviance**	1445.65	1414.96	1399.70	1400.02

*The reference category for gender was male. adjBMI, adjusted BMI; ER, maladaptive emotion regulation; $\sigma_{\mathrm{u0}}^{2}$, between-person variance; EE, trait emotional eating; $\sigma_{\mathrm{u1}}^{2}$, variance in the slope; $\sigma_{\mathrm{e0}}^{2}$, within-person variance; σ_*u0u1*_, covariance between random intercept and random slope; Null Model, random intercept-only model for hunger eating; Model 1, unconditional growth model for hunger eating. ^∗^*p* < 0.05, ^∗∗^*p* < 0.01, ^∗∗∗^*p* < 0.001.*

**Table 5:** Daily snacking as a function of stress, maladaptive emotion regulation, and trait emotional eating.

	Null Model	Model 1	Model 2	Model 3
**Fixed parameters**				
Constant	184.54 (10.22)^∗∗∗^	216.00 (12.83)^∗∗∗^	215.03 (12.94)^∗∗∗^	214.99 (12.86)^∗∗∗^
Time		-11.26 (3.04)^∗∗∗^	-52.54 (19.97)^∗∗^	-44.07 (19.18)^∗^
**Trajectories**				
Age × time			2.74 (1.43)^†^	2.18 (1.37)
Gender × time			9.48 (4.60)^∗^	7.71 (4.55)^†^
adjBMI × time			-0.28 (0.12)^∗^	-0.25 (0.12)^∗^
Stress × time			-0.24 (0.20)^∗^	-1.03 (1.21)^∗^
ER × time			-0.24 (0.20)	
Stress × ER × time			-0.01 (0.01)	
EE × time				0.47 (0.29)
Stress × EE × time				-0.01 (0.01)^†^
**Random Parameters**				
$\sigma_{\mathrm{u0}}^{2}$	5898.61 (1395.53)^∗∗∗^	5399.29 (2290.89)^∗^	5669.93 (2319.51)^∗^	5492.90 (2292.78)^∗^
σ_u0u1_		23.88 (451.49)	-272.61 (469.79)	-158.29 (454.98)
$\sigma_{\mathrm{u1}}^{2}$		45.31 (127.75)	59.80 (128.69)	27.24 (123.85)
$\sigma_{\mathrm{e0}}^{2}$	19096.32 (1275.61)^∗∗∗^	18337.40 (1347.69)^∗∗∗^	18198.59 (1336.88)^∗∗∗^	18197.71 (1336.68)^∗∗∗^
**Deviance**	6896.36	6881.55	6866.32	6865.11

*The reference category for gender was male. adjBMI, adjusted BMI; ER, maladaptive emotion regulation; EE, trait emotional eating; $\sigma_{\mathrm{u0}}^{2}$, between-person variance; $\sigma_{\mathrm{u1}}^{2}$, variance in the slope; $\sigma_{\mathrm{e0}}^{2}$ within-person variance; σ_*u0u1*_, covariance between random intercept and random slope; Null Model, random intercept-only model for snacking; Model 1, unconditional growth model for snacking. ^∗^*p* < 0.05, ^∗∗^*p* < 0.01, ^∗∗∗^*p* < 0.001; ^†^marginally significant: *p* < 0.10.*

In addition, we fitted a random intercept-only model for the independent variable stress. Again, the between-youth [$\sigma_{\mathrm{u0}}^{2}$ = 217.33, *SE* = 41.44, χ^2^(1) = 27.51, *p* < 0.001] and within-youth [$\sigma_{\mathrm{e0}}^{2}$ = 328.37, *SE* = 21.99, χ^2^(1) = 223.06, *p* < 0.001] variance components were significant. The VPC indicated that 39.83% of the variance in daily stress was situated between youth. Furthermore, the unconditional growth model for stress revealed that stress-levels generally decreased as the week progressed.

### Do Stress and Maladaptive Emotion Regulation Interact in Explaining Trajectories of Daily Desire to Eat?

To test our hypothesis, we extended the unconditional growth model for daily desire to eat (Model 1; Table 3) by adding stress and emotion regulation, as well as their interaction, to the model. Interestingly, this model (Model 2; Table 3) resulted in a significant improvement in model fit, χ^2^(6) = 18.49, *p* = 0.005. Inspection of the individual Wald test showed that daily stress significantly predict trajectories of desire to eat χ^2^(1) = 11.74, *p* < 0.001, revealing that with higher levels of stress, desire to eat showed a less steep decrease throughout the week whereas maladaptive emotion regulation χ^2^(1) = 0.95, *p* = 0.329, as well as the interaction χ^2^(1) = 0.19, *p* = 0.663 were not significantly predictive for these trajectories.

### Do Stress and Maladaptive Emotion Regulation Interact in Explaining Trajectories of Hunger Eating?

We extended the unconditional growth model for daily hunger eating (Model 1; Table 4) by adding stress and emotion regulation, as well as their interaction, to the model. Interestingly, this model (Model 2; Table 4) resulted in a significant improvement in model fit, χ^2^(6) = 15.25, *p* = 0.018. Inspection of the individual Wald test showed that daily stress significantly predicted trajectories of hunger eating χ^2^(1) = 13.02, *p* < 0.001, revealing that with higher levels of stress, hunger eating behavior showed a less steep decrease throughout the week whereas maladaptive emotion regulation χ^2^(1) = 0.33, *p* = 0.563, as well as the interaction χ^2^(1) = 0.18, *p* = 0.669 were not significantly predictive for these trajectories.

### Do Stress and Emotion Regulation Interact in Explaining Trajectories of Daily Snacking?

We extended the unconditional growth model for daily snacking (Model 1; Table 5) by adding stress and emotion regulation, as well as their interaction, to the model. Interestingly, this model (Model 2; Table 5) resulted in a significant improvement in model fit, χ^2^(6) = 15.23, *p* = 0.019. Inspection of the individual Wald test showed that daily stress χ^2^(1) = 0.87, *p* = 0.351, maladaptive emotion regulation χ^2^(1) = 1.46, *p* = 0.226, as well as the interaction χ^2^(1) = 0.37, *p* = 0.542 were not significantly predictive for these trajectories.

### Do Stress and Trait Emotional Eating Interact in Explaining Trajectories of Desire to Eat?

We extended the unconditional growth model for daily desire to eat (Model 1; Table 3) by adding stress and emotional eating, as well as their interaction, to the model. Interestingly, this model (Model 3; Table 3) resulted in a significant improvement in model fit, χ^2^(6) = 20.52, *p* = 0.002. Inspection of the individual Wald test showed that the effect of emotional eating on trajectories of desire to eat was marginally significant χ^2^(1) = 2.97, *p* = 0.085, revealing that with higher levels of emotional eating, desire to eat showed a less steep decrease throughout the week. The interaction between daily stress and emotional eating was not significant χ^2^(1) = 0.33, *p* = 0.566.

### Do Stress and Trait Emotional Eating Interact in Explaining Trajectories of Hunger Eating?

We extended the unconditional growth model for daily hunger eating (Model 1; Table 4) by adding stress and emotional eating, as well as their interaction, to the model. Interestingly, this model (Model 3; Table 4) resulted in a significant improvement in model fit, χ^2^(6) = 14.94, *p* = 0.021. Inspection of the individual Wald test showed that despite the significant effect of daily stress, emotional eating χ^2^(1) = 0.14, *p* = 0.707, as well as the interaction between stress and emotional eating, χ^2^(1) = 0.03, *p* = 0.862, was not significantly predictive for these trajectories.

### Do Stress and Trait Emotional Eating Interact in Explaining Trajectories of Daily Snacking?

We extended the unconditional growth model for daily snacking (Model 1; Table 5) by adding stress and EE, as well as their interaction, to the model. Interestingly, this model (Model 3; Table 5) resulted in a significant improvement in model fit, χ^2^(6) = 139.69, *p* < 0.001. Inspection of the individual Wald test showed that despite the significant effect of daily stress, emotional eating χ^2^(1) = 0.72, *p* = 0.396, was not significantly predictive for these trajectories. The interaction term between stress and emotional eating was only marginally significant χ^2^(1) = 2.66, *p* = 0.102.

## Discussion

The current study aimed to investigate the relationship between daily stress and eating behavior in children and adolescents (10–17 years old) using a daily diary-design. Combining a daily diary-design with the use of self-report trait questionnaires allowed us to look into the moderating role of emotion regulation and emotional eating.

First, we found that maladaptive emotion regulation was positively correlated with emotional eating, meaning emotional eating can be seen as a proxy of maladaptive emotion regulation, which is in line with Evers et al. (2010) and Michopoulos et al. (2015). Besides, daily stress was positively correlated with daily desire to eat and hunger eating; but not with daily snacking; and these three indicators of daily eating behavior were all significantly positively correlated. The positive correlation between daily stress on the one hand and desire to eat motives and hunger eating motives on the other hand was expected, as (1) the item on ‘desire to eat motives’ is seen as a desire or craving for food after experiencing stress and thus may be a way of regulating the experienced stress and (2) the item on ‘hunger to eat motives’ is seen as eating out of hunger after experiencing stress; and previous research showed that stress, operationalized as a hyperactive cortisol axis, contributes to higher levels of hunger feelings (Gluck et al., 2004; Goldschmidt et al., 2018). The correlations are in line with the reported positive associations between levels of stress and levels of desire to eat- and hunger eating motives by De Vriendt et al. (2009) in children and Reichenberger et al. (2016) in adults. In contrary to previous findings (O’Connor et al., 2008; Reichenberger et al., 2016), we could not find an association between daily stress and snacking; maybe due to specific characteristics of the study population: children instead of adults. Children and youngsters might not have the autonomy to decide what they will eat, especially snacks. Gevers et al. (2015) found that most parents both communicate about food and at the same time restrict their children on snacking behavior.

All three indicators of eating behavior showed bilateral significant positive correlations, which is in line with our expectations. Although the motivation differs in desire and hunger eating, a positive correlation is not unexpected since in both hedonic and homeostatic eating the ghrelin secretion (hunger hormone) is elevated, and the cholecystokinin-33 secretion (satiety hormone) is decreased (Monteleone et al., 2013). Concluding, variables measured on trait questionnaires (FEEL-KJ and DEBQ) correlated bilateral; and the variables measured on a daily basis (daily stress and the indicators of eating behavior) correlated with each other, but no correlations between the trait variables and daily measurements were found. For this, a potential explanation could be found in that the used questionnaires were not sensitive enough for capturing these momentary daily fluctuations.

Second, we found evidence for the hypothesis that daily stress was significantly associated with the trajectories of desire to eat and hunger eating motives, which is in line with De Vriendt et al. (2009) and Reichenberger et al. (2016). With higher levels of daily stress, desire to eat and hunger eating motives showed a less steep decrease throughout the week. As the trajectories of the eating behavior indicators decreased significantly throughout the week for every participant, the steepness of these decreases were of interest. The decreases of the hunger eating motives trajectories and of the desire to eat motives trajectories were significantly less steep in persons reporting higher levels of stress than in persons reporting lower levels of stress. This effect was not found for snacking.

Third, for both eating motives and snacking, no effect of maladaptive emotion regulation nor an interaction between daily stress and maladaptive emotion regulation was found. These findings are in contradiction with Evers et al. (2010), Vandewalle et al. (2014), and Aparicio et al. (2016). A possible explanation is the methodology of the study, as above mentioned studies are longitudinal, cross-sectional (questionnaires) or experimental studies while the current research is a diary study. Still, the moderating factor, maladaptive emotion regulation, was measured as a trait variable and therefore might not be sensitive enough to capture momentary daily effects. Next, the participants only filled out the diary during 7 days, three times a day, but only the measurement point after school was taking into account in the analysis. We could question if using one data time point a day during 7 days is enough to capture the momentary daily fluctuations. To approach these methodological shortcomings, it is recommended to include a daily measurement of emotion regulation and to include signal- or event-contingent sampling.

Fourth, we found marginally significant evidence for the hypothesis that daily stress in interaction with trait emotional eating is associated with the trajectories of desire to eat and snacking; but not for hunger eating. These results mean that in youngsters with a high emotional eating style, when experiencing high stress, a less steep decrease in desire to eat and snacking occurs in comparison with youngsters with a lower emotional eating style. These results are in line with O’Connor et al. (2008) and Reichenberger et al. (2016).

Fifth, analyses of the daily diary data also revealed that daily stress and daily eating behavior decreased as the week progressed. This was not expected and has to be explored in the future. One of the explanations can be the possibility of the contributing factor of the stress-levels fitting the day of the week. These stress-levels may play a role in the findings in this study (Areni et al., 2011; van Roekel et al., 2015). In future research, it is recommended to start the diary study on different days, to control for this order effect.

This study has several notable strengths. First, we used a daily diary design, enabling a more momentary inspection of the relation between stress and eating behavior in a naturalistic environment. Having a good reliability, validity and generalizability, diary studies are able to determine experiences, mood, behavior and contextual factors more detailed (Moskowitz and Young, 2006; Suveg et al., 2010; Engel et al., 2016; Burke et al., 2017; Goldschmidt et al., 2018). Second, we recruited a fairly large sample of youngsters between 10 and 17 years of age given that research in this age group is lacking. Yet, researching the effects of stress on wellbeing in this critical developmental period is of utmost importance given the heightened risk for developing overweight and eating pathology in particular (Giedd et al., 2009). Third, because of the highly demanding design of the study for the youngsters (diary for 7 days), a lot of efforts by the researchers were made to increase the persevered motivation of the youngsters. During a home visit the rationale was explained, a reward at the end of the study was promised and the youngsters received a personalized smartphone as incentive during the study. During the study reminders were sent for all assessing points and youngsters received a motivating phone call in between.

Despite these strengths, several limitations of the current study warrant discussion. The first limitation of the present study design is the limited number of data points each day, more specifically at breakfast, after school and before bedtime. Using signal- or event-contingent data sampling would be better to capture the daily relationship between stress and eating behavior. However, a diary-study for 7 consecutive days might be a long period for children and adolescents between 10 and 17 years old and a great burden. This was confirmed by the missing values in the diary reports. As the ability and willingness of participants are determining factors for the success of a diary study, only three diary data points were used (Moskowitz and Young, 2006; Engel et al., 2016). Besides, practical barriers prevented us from using signal- or event contingent sampling as schools did not give an authorization to fill out the diary during school time. To answer the research questions, only one measurement point a day was included in the analysis, more specifically the data point after school. First of all, two important stressors for youngsters, school and peer related stress (Sotardi, 2017) could be captured at that data point. Second, previous research showed the importance of the food availability preceding the snacking, which is higher at home (or the way to home) in contrast with a school environment. Due to the age of the participants, 10–17 years, and the possibility of having a greater food availability at home (or the way to home), the choice was made to include only this data time point here (Chopra et al., 2002; Johnson et al., 2011; Pearson et al., 2014; De Decker et al., 2017a,b). Whether emotional eating might be even more prevalent later in the evening, remains to be studied.

A second limitation of the study concerns the use of self-report measurements of eating behavior. In future research, it is recommended to use more objective methods, such as taking pictures of the snacks participants ate. This requires the use of event-contingent data sampling.

A third limitation, in addition to the one-time point, is the retrospective character of these daily measures. As mentioned above, including a signal-contingent design of the daily measures would be more suitable in order to investigate the causal daily relationship between stress and eating behavior. A fourth limitation concerns the little variance in the emotion regulation and emotional eating scores. All mean scores are in accordance with the norm scores of the questionnaires, except for maladaptive emotion regulation in boys between 12 and 18 years (Braet and Beyers, 2009; Braet et al., 2013). In future research, it might be interesting to include youngsters with overweight or obesity, as emotional eating is more prevalent among overweight and obese youngsters (Braet and Beyers, 2009). As mentioned above, a fifth limitation concerns the same start day for all participants of the diary- measures, namely on Monday. To control for week-effects, it might be better to randomize the start day for filling in the diary over the participants (Areni et al., 2011; van Roekel et al., 2015). Sixth, in this study, only intensity of stress was included as independent variable, while also the source of stress is of importance (O’Connor et al., 2008). In future research, it is recommended to include sources of daily hassles to have more specified information. Different types of stress are of interest in youngsters, e.g., Family problems; Problems with Friends; Parental Rejection; School Related Stress; Time Pressure; Concerns about Body; Romantic concerns; Sports Pressure and General self-worth (Treffers et al., 2002; O’Connor et al., 2008; Reichenberger et al., 2016). Seventh, in the current study, stress intensity was measured subjectively. As the variance within persons is rather small, the question could raise if youngsters have difficulties reporting on their stress level. In the future, we will analyze with more stringent research whether the level of the stress experience of children is related with more objective measurements, like heart rate variability. Eighth, all daily variables (e.g., stress intensity, hunger- and desire to eat motives and snacking) were measured with only one item. Although, this phenomenon is seen in different daily diary studies (O’Connor et al., 2008; Reichenberger et al., 2018), future research should use more questions to operationalize the variables.

To conclude, we found that daily stress is significantly associated with the trajectories of desire to eat and hunger eating motives. But no moderation effects of maladaptive emotion regulation were found; while emotional eating has marginally significant effects on desire to eat and snacking. In the research on stress and eating behavior, there are still a lot of gaps. The current research is a first step in reducing these gaps by investigating the underlying mechanism in the relationship between daily stress and eating behavior as a predictor of weight gain by including the moderators of emotion regulation, and more specifically emotional eating.

## Ethics Statement

This study was carried out in accordance with the recommendations of Ghent University with written informed consent from all subjects. All subjects gave written informed consent in accordance with the Declaration of Helsinki. The protocol was approved by the Ethical Committee of Ghent University.

## Author Contributions

All authors listed have made a substantial, direct and intellectual contribution to the work, and approved it for publication.

## Conflict of Interest Statement

The authors declare that the research was conducted in the absence of any commercial or financial relationships that could be construed as a potential conflict of interest.
